# Supplementary material for: Sensitivity to sequencing depth in single-cell cancer genomics
Source: Genome Med. 2018 Apr 16;10:29. doi: 10.1186/s13073-018-0537-2 (PMC5901877; doi:10.1186/s13073-018-0537-2)
Supplement: Supplementary file 1 — Supplementary note containing all information required to generate the results presented in this manuscript. Tables S1 and S2. Figures S1–S3. (PDF 483 kb) [file 13073_2018_537_MOESM1_ESM.pdf]

Supplementary Note

Sensitivity to sequencing depth in single-cell cancer genomics

João MF Alves & David Posada

Department of Biochemistry, Genetics and Immunology, University of Vigo, Spain.
Biomedical Research Center (CINBIO), University of Vigo, Spain.
Galicia Sur Health Research Institute, Vigo, Spain.

Introductory note

In the following supporting document, we will go through all steps required to generate the results presented in the main manuscript. For reproducibility purposes, this guideline contains all information needed (e.g., software tools, command line arguments, etc.) to generate the obtained results. Please note that all code chunks that begin with **'$'** are to be run in the shell while the ones without it should be run in **R**. Importantly, while we restrict this step-by-step tutorial to the data from the Ni *et al.* (2013) study, as this corresponds to the smallest dataset available, the very same pipeline was applied to the data from the remaining studies.

---

1. Data collection and overview

The following publicly available NGS datasets from four distinct single-cell sequencing (SC-Seq) studies were retrieved from the Sequence Read Archive (SRA):

- **1)** Ni *et al.* (2013) PNAS:
  → 8 Circulating Tumor Cells (CTCs) from one lung adenocarcinoma patient (P1).
- **2)** Xu *et al.* (2012) Cell:
  → 25 Single cells derived from the primary tumor of a single kidney tumor patient.
- **3)** Wang *et al.* (2014) Nature:
  → 59 (4 WGS + 55 WXS) Single cells derived from the primary tumor of a single breast cancer patient.
- **4)** Hou *et al.* (2012) Cell:
  → 65 Single cells derived from a single JAK-2 negative neoplasm myeloproliferative patient.

1.1 Downloading through the SRA

Load the **R** packages needed and use *SRAdb* to retrieve the deposited data. Please note that we will be using $HOME as our default storage and working directory throughout this entire guideline.

```
# Try http:// if https:// URLs are not supported
source("https://bioconductor.org/biocLite.R")
biocLite("SRAdb")
library(SRAdb)

# Download and uncompress metadata file
sqlfile <<- getSRAdbFile()
sra_con = dbConnect(SQLite(), sqlfile)

# Load list of SRRNames of interest to download
Nietal.SRRNAmes <- read.table('$HOME/Nietal.P01.SRRNames', sep='\t', head=F)
Nietal.SRRNAmes
          V1      V2
1  SRR975190 P01C01E
2  SRR975192 P01C02E
3  SRR975194 P01C03E
4  SRR975196 P01C04E
5  SRR975198 P01C05E
6  SRR975200 P01C06E
7  SRR975202 P01C07E
8  SRR975204 P01C08E
9  SRR975206 P01D01E
10 SRR975210 P01M01E
11 SRR975212 P01P01E

for (i in 1:dim(Nietal.SRRNames)[1]) {
getSRAfile(Nietal.SRRNames[i,1], sra_con, fileType='sra', destDir="$HOME")
}
```

Rename the downloaded files to the sample names provided in Ni *et al.* (2013), and run the *sratoolkit* to convert NCBI SRA (.sra) format into FASTQ:

```
$ for i in *.sra
    do
    SRA_NAME=$(echo "${i%.*}")
    SAMPLE_NAME=$(grep $SRA_NAME Nietal.P01.SRRNames | cut -f 2)
    mv "$SRA_NAME".sra "$SAMPLE_NAME".sra

    fastq-dump "$SAMPLE_NAME".sra --split-3 --gzip -O "$HOME"
    done
```

1.2 FASTQ alignment & pre-call processing

Align raw FASTQs using *BWA* **mem** algorithm described in Li (2013) and convert them into BAM files using the *Samtools* software.

```
$ NbSamples=$(wc -l Nietal.P01.SRRNames | cut -f 1 -d " ")
$ for ((i=1; i<=NbSamples; i++))
    do
    Sample=$(sed "${i}q;d" Nietal.P01.SRRNames | cut -f 2)
    bwa mem -t 12 -M \
      -R "@RG\tID:${Sample}\tSM:${Sample}\tPL:Illumina\tLB:${Sample}\tPU:HiSeq2000_2500" \
      hs37d5.fa "${Sample}_1.fastq.gz" "${Sample}_2.fastq.gz" | \
      samtools view -Sbq 20 -@ 12 - > "${Sample}.bam"
    done
```

Mark possible PCR duplicates using *Picard*.

```
$ for i in *bam
    do
    SAMPLE_NAME=$(echo "${i%.*}")
    picard MarkDuplicates \
      INPUT="$SAMPLE_NAME".bam \
      OUTPUT="$SAMPLE_NAME".Dedup.bam \
      METRICS_FILE="$SAMPLE_NAME".Dedup.metrics \
      CREATE_INDEX=true \
      TMP_DIR=$HOME \
      VALIDATION_STRINGENCY=LENIENT
```

The command line above will create a sorted BAM file called *$SAMPLE_NAME*.Dedup.bam with the same content as the input file, except that any duplicate reads are marked as such. Note that reads that *Picard* marks as duplicates do not necessarily have identical sequence they just map to the same chromosomal location.

Perform local realignment around indels with the *Genome Analysis ToolKit* (short: GATK). **Please note that the next steps (until 2.2) should only be run for single-cell data. Below, we explain why.**

```
$ for i in *.Dedup.bam
  do
  SAMPLE_NAME=$(echo "${i%%.*}")
  GenomeAnalysisTK -T RealignerTargetCreator \
    -R hs37d5.fa \
    -I "$SAMPLE_NAME".Dedup.bam \
    -o "$SAMPLE_NAME".Dedup.list \
    -known Mills_and_1000G_gold_standard.indels.b37.vcf \
    -nt 12

  GenomeAnalysisTK -T IndelRealigner \
    -R hs37d5.fa \
    -I "$SAMPLE_NAME".Dedup.bam \
    -o "$SAMPLE_NAME".Dedup.Real.bam \
    -targetIntervals "$SAMPLE_NAME".Dedup.list \
    -known Mills_and_1000G_gold_standard.indels.b37.vcf
  done
```

Recalibrate the base quality scores (for improved SNP calling) using *GATK's BaseRecalibrator*:

```
$ for i in *.Dedup.Real.bam
  do
  SAMPLE_NAME=$(echo "${i%%.*}")
  GenomeAnalysisTK -T BaseRecalibrator \
    -R hs37d5.fa \
    -I "$SAMPLE_NAME".Dedup.Real.bam \
    -o "$SAMPLE_NAME".Dedup.Real.table \
    -knownSites dbsnp_138.b37.vcf \
    -nct 12

  GenomeAnalysisTK -T PrintReads \
    -R hs37d5.fa \
    -I "$SAMPLE_NAME".Dedup.Real.bam \
    -o "$SAMPLE_NAME".Dedup.Real.Recal.bam \
    -BQSR "$SAMPLE_NAME".Dedup.Real.table \
    -nct 12
  done
```

1.3 Downsampling single-cell BAMs to pre-defined depths

In the Ni *et al.* (2013) study, each single-cell was sequenced using Illumina technology to around 47x coverage. *Picard* can be used to downsample the original data to different depths. For statistical validation, 10 replicates per single-cell (e.g., 10 P01C01E cells downsampled to 25x, 10x, 5x and 1x) will be generated using the following arguments:

Table I. Downsample fractions for Ni *et al.* (2013) dataset

Depth	Ni et al. (2013)
**1x**	0.0210
**5x**	0.1050
**10x**	0.2100
**25x**	0.5251
**Original (X)**	47.61

```
# Here one should only use the single-cell BAMs files
$ for i in *.Dedup.Real.Recal.bam
    do
    SAMPLE_NAME=$(echo "${i%%.*}")

      for j in  {1..10}
      do
      picard DownsampleSam \
      INPUT="$SAMPLE_NAME".Dedup.Real.Recal.bam \
      OUTPUT="$SAMPLE_NAME".1x.REP$j.bam \
      RANDOM_SEED=$j \
      PROBABILITY=0.0210
      done
    done
```

To visualize the effects of downsampling on sequencing coverage, *bedtools* can be used to estimate the breadth and depth of coverage of the downsampled SC-Seq data (***Figure 1 of Main Text***).

2. Variant Calling

2.1 Single-Cell variant calling using *MONOVAR*

For variant calling, the *Monovar* algorithm, which was specifically designed for SC-Seq data, will be used:

```
# 1. Create text files (one for each of the specified depths) with single-cell BAMs to analyze.
# Here's an example for the 1x set:
$ cat $HOME/Nietal.2013.1x.Paths
    $HOME/P01C01E.Final.1x.REP${i}.bam
    $HOME/P01C02E.Final.1x.REP${i}.bam
    $HOME/P01C03E.Final.1x.REP${i}.bam
    $HOME/P01C04E.Final.1x.REP${i}.bam
    $HOME/P01C05E.Final.1x.REP${i}.bam
    $HOME/P01C06E.Final.1x.REP${i}.bam
    $HOME/P01C07E.Final.1x.REP${i}.bam
    $HOME/P01C08E.Final.1x.REP${i}.bam

# 2. Set MONOVAR Run for all 10 Replicates:
$ for i in  {1..10}
  do
  sed -e "s/\${i}/$i/" $HOME/Nietal.2013.1x.Paths > $HOME/"Nietal.2013.1x.REP${i}"
  samtools mpileup -BQ0 -d10000 -f hs37d5.fa -q 40 \
    -b $HOME/"Nietal.2013.1x.REP${i}" | monovar.py -p 0.002 -a 0.2 -t 0.05 \
    -m 4 -f hs37d5.fa -b /$HOME/"Nietal.2013.1x.REP${i}" -o $HOME/"Nietal.2013.1x.REP${i}.vcf"
  done
```

2.2 Bulk-level variant calling using *VarDict*

Population-level (i.e., bulk) variants will be called using the *VarDict* software under a paired-sample variant calling approach. As noted above, the de-duplicated bulk BAMs will be used as input, as *VarDict* performs local realignment internally. Since Ni *et al.* (2013) obtained both primary tumor and metastatic samples, two independent runs of *VarDict* are needed: Normal *vs.* Primary Tumor, and Normal *vs.* Metastasis, respectively. Below we only show the example for the primary tumor sample. The same strategy should be followed for the metastatic sample. Both calls can be then combined using *GATK* (i.e., *CombineVariants*):

```
# Here, we only need our bulk samples and the bed file with the specific exome targets.
# Perform variant calling for the primary tumor sample:
$ AF_THR="0.01"
$ VarDict -th 1 -G hs37d5.fa -f $AF_THR -N P01P01E \
    -b "$HOME/P01P01E.Dedup.bam|$HOME/P01D01E.Dedup.bam" -c 1 -S 2 -E 3 -q 25 \
    -g 4 $HOME/SureSelect_ExomeTargets_GRCh37.bed | testsomatic.R | var2vcf_paired.pl \
    -N "P01P01E|P01D01E" -f $AF_THR -P 0.9 -m 4.25 > $HOME/Nietal.2013.BULK.PrimaryTumor.vcf
```

---

3. Sensitivity towards variants detection

3.1 Gold-standard SNV Set from bulk

After combining the results from the Primary Tumor and Metastasis, we will filter the Ni *et al.* (2013) bulk-level VCF to only include **Germline** and **Somatic** point-mutations by combining *GATK*'s SelectVariants tool together with *bcftools*:

```
# 1. Get all good-quality SNVs detected by VarDict
$ GenomeAnalysisTK -T SelectVariants
    -R hs37d5.fa \
    -V $HOME/Nietal.2013.BULK.PT_Met.vcf \
    -selectType SNP \
    -ef \
    --out $HOME/Nietal.2013.BULK.PT_Met.Pass-SNVs.vcf

# 2. Keep germline variants detected by VarDict using the STATUS field printed in VCF
$ bcftools filter -i 'STATUS == "Germline"' $HOME/Nietal.2013.BULK.PT_Met.Pass-SNVs.vcf \
    -o $HOME/Nietal.2013.BULK.PT_Met.Pass-Germline.SNVs.vcf

# 3. Use the same strategy to only include somatic calls
$ bcftools filter -i 'STATUS == "StrongSomatic" || STATUS == "LikelySomatic"' \
    $HOME/Nietal.2013.BULK.PT_Met.Pass-SNVs.vcf \
    -o $HOME/Nietal.2013.BULK.PT_Met.Pass-Somatic.SNVs.vcf
```

3.2 Sensitivity of single-cell callsets towards (bulk-level) variants detection

VCFtools can be used to estimate the amount of gold-standard SNVs present in single-cell calls:

```
# Example for the 1x set:
$ for i in  {1..10}
  do
  vcftools --vcf $HOME/"Nietal.2013.1x.REP${i}.vcf"
    --positions $HOME/Nietal.2013.BULK.PT_Met.Pass-Germline.SNVs.list --recode \
    --out $HOME/"Nietal.2013.1x.REP${i}.Germline"

  vcftools --vcf $HOME/"Nietal.2013.1x.REP${i}.vcf"
    --positions $HOME/Nietal.2013.BULK.PT_Met.Pass-Somatic.SNVs.list --recode \
    --out $HOME/"Nietal.2013.1x.REP${i}.Somatic"
  done
```

Barplots can be used to illustrate the proportion of bulk-level variants across all downsampling experiments (***Figure 2.A & 2.B of Main Text***).

3.3 Quantifying somatic precision in SC-Seq experiments

Besides estimating bulk-level variants recall, it is also important to quantify the fraction of SNVs found in the down-sampled replicates that are also identified in the original single-cell datasets (i.e., somatic precision). This can be done using *GATK's VariantEval* (***Figure 2.C of Main Text***):

```
# 1. Run VariantEval for all SC VCFs
$ for i in {1..10}
    do
    java -jar $GATK -T VariantEval -R hs37d5.fa \
    --eval:"Nietal.1x.REP${i}" $HOME/"Nietal.2013.1x.REP${i}.Somatic.recode.vcf" \
    --eval:"Nietal.5x.REP${i}" $HOME/"Nietal.2013.5x.REP${i}.Somatic.recode.vcf" \
    --eval:"Nietal.10x.REP${i}" $HOME/"Nietal.2013.10x.REP${i}.Somatic.recode.vcf" \
    --eval:"Nietal.25x.REP${i}" $HOME/"Nietal.2013.25x.REP${i}.Somatic.recode.vcf" \
    --comp "Nietal.2013.Original.Somatic.vcf" \
    -o $HOME/"Nietal.2013.REP${i}.Recall"
  done
```

```
# 2. Use table to print proportions
$ NbOriginal=$(grep "ValidationReport" $HOME/"Nietal.2013.REP${i}.Recall" | \
               grep "all" | tail -n 1 | awk 'BEGIN{FS=" "}{print $6}')
$ for i in {1..10}
  do
  echo -e "Dataset\tReplicate\tProportionOfOverlap\tProportionOfRecall" \
  > $HOME/"Nietal.2013.REP${i}.Recall.Table"
  grep "CompOverlap" $HOME/"Nietal.2013.REP${i}.Recall" | \
  grep "all" | sed 's/ \+ /\t/g' | cut -f 3,9 | awk 'BEGIN{FS="\t"}{print $1"\tREP"'$i'"\t"$2}'
  >> $HOME/"Nietal.2013.REP${i}.Recall.Table"
  done
```

3.4 Quantifying the proportion of single-cell specific variants (i.e., bulk-absent)

Below we will quantify the proportion of SNVs found exclusively in the single-cell datasets (***Figure S1***):

```
# 1. Merge bulk-level positions
$ cat $HOME/Nietal.2013.BULK.PT_Met.Pass-Germline.SNVs.list \
  $HOME/Nietal.2013.BULK.PT_Met.Pass-Somatic.SNVs.list \
  > $HOME/Nietal.2013.BULK.ALL.list

# 2. Filter out bulk positions and low quality sites and count each variant "type"
$ for j in 1x 5x 10x 25x
  do
    for i in {1..10}
    do
    vcftools --vcf $HOME/"Nietal.2013.$j.REP${i}.vcf" \
    --exclude-positions  $HOME/Nietal.2013.BULK.ALL.list --remove-indels --remove-filtered-all \
    --recode --out $HOME/"Nietal.2013.$j.REP${i}.ConsensusCall"


nb_Somatic=$(grep -v "#" $HOME/"Nietal.2013.$j.REP${i}.Somatic.recode.vcf" | wc -l)
nb_Germline=$(grep -v "#" $HOME/"Nietal.2013.$j.REP${i}.Germline.recode.vcf" | wc -l)
nb_Census=$(grep -v "#" $HOME/"Nietal.2013.$j.REP${i}.ConsensusCall" | wc -l)
nb_Rep=$(echo $i REP$j)
bulk=$(echo "scale=5 ; $nb_Somatic + $nb_Germline" | bc)
total=$(echo "scale=5 ; $nb_Somatic + $nb_Germline + $nb_Census" | bc)
echo $nb_Rep $bulk $nb_Census $total >> $HOME/Nietal.2013.Bulk-shared_SC-specific.Counts

done
done
```

4. Characterizing *driver* alterations in cancer genomes from SC-Seq data

4.1 Overlap with COSMIC database

In order to quantify the proportion of COSMIC variants preserved across sequencing depths, COSMIC coding mutations need to be retrieved from the ***COSMIC FTP website*** in VCF format. Using *VCFTools*, the COSMIC variants listed in the reference callset can be compared against the downsampled calls as follows:

```
# 1. Get intersect between COSMIC and reference callset
$ bgzip $HOME/Nietal.2013.Original.Somatic.vcf
$ bgzip $COSMIC/CosmicCodingMuts.vcf
$ tabix -p vcf $HOME/Nietal.2013.Original.Somatic.vcf.gz
$ tabix -p vcf $COSMIC/CosmicCodingMuts.vcf.gz
$ vcf-isec -f -n +2 $HOME/Nietal.2013.Original.Somatic.vcf.gz $COSMIC/CosmicCodingMuts.vcf.gz \
  > $HOME/Nietal.2013.Original.Cosmic.vcf

# 2. Get proportion of COSMIC variants conserved (vcf-isec can also be used)
$ grep -v "#" $HOME/Nietal.2013.Original.Cosmic.vcf | cut -f 1,2 > $HOME/Nietal.2013.COSMIC.list
$ for i in 1x 5x 10x 25x
  do
    for j in  {1..10}
    do
    vcftools --vcf $HOME/"Nietal.2013.$i.REP${j}.Somatic.recode.vcf" \
      --positions $HOME/$HOME/Nietal.2013.COSMIC.list \
      --recode \
      --out $HOME/"Nietal.2013.$i.REP${j}.Somatic.COSMIC"

    nb_SomaticCOSMIC=$(grep -v "#" $HOME/"Nietal.2013.$i.REP${j}.Somatic.COSMIC.recode.vcf" | wc -l)
    Nietal_nb=$(wc -l $HOME/$HOME/Nietal.2013.COSMIC.list | awk 'BEGIN{FS=" "}{print $1}')
    Proportion=$(echo "scale=5 ; $nb_SomaticCOSMIC / $Nietal_nb" | bc)
    nb_Rep=$(echo $i REP$j)
    echo $nb_Rep $Proportion >> $HOME/Nietal.2013.COSMICpreserved
    done
  done
```

4.2 Characterizing previously described non-synonymous events

Using the variant-calling pipeline described above, 29 non-synonymous mutations (*out of the 54, originally reported by the authors*) have been detected in the reference callset. Using this set of mutations, we'll next evaluate the extent to which sequencing depth affects the detection of *driver alterations* by measuring the proportion of such non-synonymous variants in the downsampling experiments. Importantly, this also represents a good approach to simultaneously benchmark the variant calling strategy.

```
$ for i in 1x 5x 10x 25x
  do
    for j in  {1..10}
    do
    vcftools --vcf $HOME/"Nietal.2013.$i.REP${j}.Somatic.recode.vcf" \
      --positions $HOME/Nietal.2013.NonSynonymous.list \
      --recode \
      --out $HOME/"Nietal.2013.$i.REP${j}.Somatic.NonSyn"

    nb_SomaticNonSyn=$(grep -v "#" $HOME/"Nietal.2013.$i.REP${j}.Somatic.NonSyn.recode.vcf" | wc -l)
    Nietal_nb=$(wc -l $HOME/Nietal.2013.NonSynonymous.list | awk 'BEGIN{FS=" "}{print $1}')
    Proportion=$(echo "scale=5 ; $nb_SomaticNonSyn / $Nietal_nb" | bc)
    nb_Rep=$(echo $i REP$j)
    echo $nb_Rep $Proportion >> $HOME/Nietal.2013.NbNonSynonymous
    done
  done
```

Bar- and tile-plots can then be used to illustrate the results between the different downsampling sets for both **COSMIC** and **non-synonymous** variants conserved (***Figure 3 of Main Text***).

---

5. Genotype concordance rates across sequencing depth

Genotype concordance rates can be estimated by comparing the genomic profiles from the reference callset with the ones obtained from our downsampling experiments.

```
$ echo -e "Study\tSet\tReplicate\tConcord\tDiscord\tMissing" > Results.Nietal.2013.1x.GenoConc.txt
$ for i in {1..10}
  do

  # Transform VCF into genotype matrix
  vcftools --vcf "Nietal.2013.1x.REP${i}.Somatic.recode.vcf" --extract-FORMAT-info GT \
    --out "Nietal.2013.Somatic.1x.REP${i}"

  # Get positions that overlap original callset
  cut -f 1,2 "Nietal.2013.Somatic.1x.REP${i}.GT.FORMAT" > Positions.txt
  fgrep -w -f positions.txt Nietal.2013.Somatic.Original.GT.FORMAT > Original.Comp.txt

  # Change format to ternary format
  cat Original.Comp.txt | sed 's/0\/0/0/g' | \
  sed 's/0\/1/1/g' | sed 's/1\/1/1/g' | sed 's/\.\/\./3/g' > Original.Comp.treated.txt
  cat "Nietal.2013.Somatic.1x.REP${i}.GT.FORMAT" | \
  sed 's/0\/0/0/g' | sed 's/0\/1/1/g' | sed 's/1\/1/1/g' | sed 's/\.\/\./3/g' > Downsamp.treated.txt
  awk '{print $1 "_" $2"\t"$0}' Downsamp.treated.txt | cut -f 1,4- > Downsamp.treated.f.txt
  awk '{print $1 "_" $2"\t"$0}' Original.Comp.treated.txt | cut -f 1,4- > Original.Comp.treated.f.txt

  # Melt dataset to ease comparison
  ./melt.sh < Original.Comp.treated.f.txt | \
    grep -v "Pos:CHROM_POS" > Original.Comp.treated.final
  ./melt.sh < Downsamp.treated.f.txt | grep -v "Pos:CHROM_POS" > Downsamp.treated.final

  # Paste files and count differences
  paste Original.Comp.treated.final Downsamp.treated.final | \ sed 's/ /\t/g' | \
  awk 'BEGIN{FS="\t"}{if ($2==$4) print $0"\t0"; else if($2=="0"&&$4=="3"||$2=="1"&&$4=="3") \
  print $0"\t3"; else print $0"\t1"}' | \
  cut -f 5 | sort | uniq -c > Result.txt
  Total=$(awk '{ sum += $1 } END { print sum }' Result.txt)
  Conc=$(sed -n 1p Result.txt | awk 'BEGIN{FS=" "}{print $1}')
  Disc=$(sed -n 2p Result.txt | awk 'BEGIN{FS=" "}{print $1}')
  Missing=$(sed -n 3p Result.txt | awk 'BEGIN{FS=" "}{print $1}')
  ConcProp=$(echo $Conc/$Total | bc -l)
  DiscProp=$(echo $Disc/$Total | bc -l)
  MissProp=$(echo $Missing/$Total | bc -l)
  echo -e "Nietal2013\t1x\tREP"$i"\t"$ConcProp"\t"$DiscProp"\t"$MissProp \
  >> Results.Nietal.2013.1x.GenoConc.txt

  # Remove all temp files
  rm Positions.txt
  rm Original.Comp.txt
  rm Original.Comp.treated.txt
  rm Downsamp.treated.txt
  rm Downsamp.treated.f.txt
  rm Original.Comp.treated.f.txt
  rm Original.Comp.treated.final
  rm Downsamp.treated.final
  rm Result.txt

done
```

While the code above only provides genotype concordance rates for SNVs (***Figure 4 of Main Text***), the same approach can be used to estimate CNV genotype concordance rates (i.e., breakpoint conservation) (***Figure 5.B of Main Text***).

---

6. Copy-number variant conservation across sequencing depths in SC-Seq data

Aside from SNVs, copy-number variants (i.e., CNVs) are also a prevalent source of genetic variation in cancer cells. On this basis, the *GINGKO* CNV-calling algorithm can be used to explore the extent to which sequencing depth affects copy-number detection from SC-Seq data. Here, a stand-alone version of *GINKGO* was applied to all BAMs following the software recommendations:

```
# 1. Create a list of BAM files to use as input such as the one below
$ cat Nietal.2013.1x.SC.List
  P01C01E.Final.1x.REP$i.bam
  P01C02E.Final.1x.REP$i.bam
  P01C03E.Final.1x.REP$i.bam
  P01C04E.Final.1x.REP$i.bam
  P01C05E.Final.1x.REP$i.bam
  P01C06E.Final.1x.REP$i.bam
  P01C07E.Final.1x.REP$i.bam
  P01C08E.Final.1x.REP$i.bam

# 2. Generate BED files from the previous BAMs:
$ for rep in {1..10}
  do
  sed -e "s/\$i/$rep/g" Nietal.2013.1x.SC.List \
  >  "Nietal.2013/Nietal.P01.1x.REP"$rep

  while read p
  do
   i=$(basename $p); j=${i%.*}
     bedtools bamtobed -i $p > $j".bed"; done < "Nietal.P01.1x.REP"$rep
  done

# 3. Create folder to store results. Change config file accordingly and move to folder. Run GINKGO.
# Here's an example for one replicate:
$ mkdir Nietal.2013.1x.REP1
$ ls $HOME | grep .bed$ > $HOME/Nietal.2013.1x.REP1/Nietal.List
$ cp $TOOLS/GINKGO/config $HOME/Nietal.2013.1x.REP1/
$ $TOOLS/GINGKO/scripts/analyze.sh $HOME/Nietal.2013.1x.REP1/
```

While *GINKGO* outputs multiple results, we will focus on the SegBreaks file. For each genomic segment, *GINKGO* reports the presence/absence profiles of CN events for each single-cell. Using this information, we can compare the results for the reference callset against the downsampling experiments to estimate the proportion of CNVs preserved for increasing degrees of downsampling (***Figure 5.A of Main Text***).

7. Dissecting clonal populations and evolutionary history of cancer using SC-Seq data

7.1 Clonal genotypes and cluster assignment

Througout this section, the somatic SNVs that have been identified above (**section 3**) will be used to infer clonal populations from SC-Seq data. For that, the previously published *Single-Cell Genotyper* tool (*SCG*) will be applied to infer clonal populations (i.e., clusters). Since we're analysing "**real**" cancer data (and for real data we rarely - if ever - know the truth) it should be highlighted that we are not measuring accuracy towards clonal structure prediction. Rather, we are interested in exploring whether the inferences differ with respect to sequencing depth (i.e., consistency).

```
# Here's an example for the 1x set:
# You'll need to convert the VCF into a binary/ternary matrix (but see SCG manual)
# 1. Set configuration template (*config.yaml)
$ cat $HOME/SCG/Nietal.2013.1x.config.yaml
num_clusters: 40
alpha_prior: [9, 1]
kappa_prior: 1
data:
  snv:
    file:  $HOME/"Nietal.2013.1x.REP${i}.Somatic.SCG.txt.gz"
    gamma_prior: [[98, 1, 1],
                  [25, 50, 25],
                  [1, 1, 98]]
    state_prior: [1, 1, 1]

# 2. Config file replacement and SCG search-phase using doublet-aware model
$ for j {1..10}
  do
  sed -e "s/\${i}/$j/" $TOOLS/SCG/Nietal.2013.1x.config.yaml \
    > $HOME/SCG/"Nietal.2013.1x.REP${j}.config.yaml"
  for r in {1..1000}
    do
    seed=$RANDOM
    $TOOLS/SCG/scg run_doublet_model --config_file $HOME/SCG/"Nietal.2013.1x.REP${j}.config.yaml" \
      --state_map_file $TOOLS/SCG/state_map.yaml \
      --max_num_iters 20 --seed $seed  \
      --lower_bound_file $HOME/"Nietal.2013.1x.REP${j}.LB"

    res=$(cat $HOME/"Nietal.2013.1x.REP${j}.LB" | tail -n 1 | awk 'BEGIN{FS=": "}{print $2}')
    echo $seed $res >> $HOME/"Nietal.2013.1x.REP${j}.LowerBounds.txt"
    done
  done

# 3. Re-run SCG for best model, store and re-name output

$ for j in {1..10}
  do
  seed=$(sort -k2n,2 $HOME/"Nietal.2013.1x.REP${j}.LowerBounds.txt" | \
      tail -1 | awk 'BEGIN{FS=" "}{ print $1}')
  $TOOLS/SCG/scg run_doublet_model --config_file $TOOLS/SCG/"Nietal.2013.1x.REP${j}.config.yaml"
    --state_map_file $TOOLS/SCG/state_map.yaml \
    --max_num_iters 20 --seed $seed \
    --lower_bound_file $HOME/"Nietal.2013.1x.REP${j}.LB" \
    --out_dir $HOME/SCG/RESULTS/

  done
```

The "**adjusted Rand-Index**", which measures the similarity between two data clusterings, can then be used to compare the clustering consistency across datasets. This can be done in R and illustrated using simple barplots. As highlighted in the main text, a single clonal population was inferred for the Ni *et al.* dataset at all sequencing depths. Consequently, the *adjusted Rand-Index* estimated was 1 for all comparisons (***Figure 6 of Main Text** -*

only displays the variable datasets).

7.2 Clonal lineage Trees

Evolutionary lineage trees for all sequencing depths can be inferred using the *oncoNEM* R package. Following a similar approach to Ross et al. (2016), the pairwise cell shortest-path distance can be estimated between clonal lineage trees from downsampling experiments and the lineage tree obtained from the reference callset in order to measure the consistency in evolutionary reconstruction (***Figure 7 of Main Text***).

```
# 1. Convert VCF to oncoNEM binary format
$ cat $HOME/Nietal.2013.1x.REP1.oncoNEM
  cell_id P01C01E P01C02E P01C03E P01C04E P01C05E P01C06E P01C07E P01C08E
  1:35824705 1 0 1 0 1 1 1 0
  1:152128054 1 2 2 2 1 0 2 1
  1:152129094 1 0 2 0 2 1 0 1
  1:204378887 1 1 1 1 1 0 2 0
  2:168103863 1 1 0 1 1 1 1 2
  2:168103925 2 0 0 1 0 1 1 2
  2:179423113 1 0 2 2 1 1 2 1
  (...)

# 2. Load libraries required in R and datasets
library(oncoNEM)
library(igraph)
Nietal.Original <- as.matrix(read.table("$HOME/Nietal.2013.Original.oncoNEM", head=T, \
                                        + row.names=1, check.names=F))

# 2.1 Estimate FPR and FNR from data:
Fpr <- seq(from = 0.15, to = 0.3, length.out = 6)
Fnr <- seq(from = 0.05, to = 0.2, length.out = 6)
llh.Nietal.Original <- matrix(0,nrow = length(Fpr),ncol = length(Fnr))
for (i.fpr in 1:length(Fpr)) {
  for (i.fnr in 1:length(Fnr)) {
    oNEM.Nietal.Original <- oncoNEM$new(Data=Nietal.Original, FPR=Fpr[i.fpr], FNR = Fnr[i.fnr])
    oNEM.Nietal.Original$search(delta = 100)
    llh.Nietal.Original[i.fpr,i.fnr] <- oNEM.Nietal.Original$best$llh
    }
}
indx.Nietal.Original <- which(llh.Nietal.Original==max(llh.Nietal.Original), arr.ind=TRUE)

# 2.2 Get best FPR and FNR from llh:
Fpr.est.Nietal.Original <- Fpr[indx.Nietal.Original[1]]
Fnr.est.Nietal.Original <- Fnr[indx.Nietal.Original[2]]

# 2.3 Run oncoNEM with best llh values:
oNEM.Nietal.Original  <- oncoNEM$new(Data = Nietal.Original,
    FPR = Fpr.est.Nietal.Original,
    FNR = Fnr.est.Nietal.Original)
oNEM.Nietal.Original$search(delta = 200)

# 2.4 Expand model to identify any unobserved subpopulations:
oNEM.Nietal.Original.expanded <- expandOncoNEM(oNEM.Nietal.Original,epsilon = 10,delta = 200,
    checkMax = 10000,app = TRUE)

# 2.5 Cluster cells into subpopulations:
oncoTree.Nietal.Original <- clusterOncoNEM(oNEM = oNEM.Nietal.Original.expanded,
    epsilon = 10)

# 2.6 Relable cells to make sets comparable:
oncoTree.Nietal.Original$clones <- relabelCells(clones=oncoTree.Nietal.Original$clones,
                                              + labels=colnames(Nietal.Original))

# 2.7 Repeat the pipeline to downsampling data
```

```
# 2.8 Estimate pairwise cell shortest-path distances using the Original tree as reference:
Distance.Nietal.1x <- matrix(ncol=1, nrow=10)
Distance.Nietal.5x <- matrix(ncol=1, nrow=10)
Distance.Nietal.10x <- matrix(ncol=1, nrow=10)
Distance.Nietal.25x <- matrix(ncol=1, nrow=10)
for (i in 1:10){
Distance.Nietal.1x[i,1] <- treeDistance(tree1=paste("oncoTree.Nietal.1x.REP", i, sep=""),
                                        + tree2=oncoTree.Nietal.Original)
Distance.Nietal.5x[i,1] <- treeDistance(tree1=paste("oncoTree.Nietal.5x.REP", i, sep=""),
                                        + tree2=oncoTree.Nietal.Original)
Distance.Nietal.10x[i,1] <- treeDistance(tree1=paste("oncoTree.Nietal.10x.REP", i, sep=""),
                                        + tree2=oncoTree.Nietal.Original)
Distance.Nietal.25x[i,1] <- treeDistance(tree1=paste("oncoTree.Nietal.25x.REP", i, sep=""),
                                        + tree2=oncoTree.Nietal.Original)
}
```

7.3 Tumor phylogenies from SC-Seq data

Finally, the *SiFit* software will be applied to reconstruct maximum-likelihood phylogenetic trees from SC-Seq data across all sequencing depths.

```
# 1. Convert VCF to SiFit binary format
$ cat $HOME/Nietal.2013.1x.REP1.SiFit
  1_35824705 1 0 1 0 1 1 1 0
  1_152128054 1 3 3 3 1 0 3 1
  1_152129094 1 0 3 0 3 1 0 1
  1_204378887 1 1 1 1 1 0 3 0
  2_168103863 1 1 0 1 1 1 1 3
  2_168103925 3 0 0 1 0 1 1 3
  2_179423113 1 0 3 3 1 1 3 1
  2_179612324 1 0 1 3 1 0 1 1
  (...)

# 2. Set variables and run SiFit
$ for j {1..10}
  do
  nbcells=$(awk '{print NF}' $HOME/"Nietal.2013.1x.REP{$j}.SiFit" | sort -nu | awk '{print $0-1}')
  nbMuts=$(wc -l $HOME/"Nietal.2013.1x.REP{$j}.SiFit" | awk 'BEGIN{FS=" "}{print $1}')
  java -jar $TOOLS/SiFit/SiFit.jar -m $nbcells -n $nbMuts -fp 0.02 -fn 0.2 -iter 200000 -df 0 \
      -ipMat $HOME/"Nietal.2013.1x.REP{$j}.SiFit" \
      > $HOME/"Nietal.2013.1x.REP{$j}.SiFit.Results"
  tail -n 1 $HOME/"Nietal.2013.1x.REP{$j}.SiFit.Results" | awk 'BEGIN{FS=" "}{ print $4}' \
      > $HOME/"Nietal.2013.1x.REP{$j}.SiFit.Tree.newick"
  done
```

SiFit directly outputs a tree in *newick* format. The resulting trees can thus be fed into *phangorn* R package in order to estimate the Robinson-Foulds distance - which measures the topological distance between phylogenies - between the "downsampling trees" and the phylogenetic tree obtained from the reference callset (***Figure 8 of Main Text***), and the Homoplasy Index (CI) estimates of each tree - a measure of the amount of homoplasy on a tree; The HI is one minus the ratio between the minimum number of changes required and the actual number observed (***Figure S3***).

Supplementary Tables

Table SI. Overview of SC-Seq data used in the current study

CellID	Mean Depth	1x Breadth	*Study*	*Type*	SRA ID
P1-CTC1	49,94	0,77	*Ni et al. (2013)*	*WXS*	*SRP029757*
P1-CTC2	43,99	0,59	*Ni et al. (2013)*	*WXS*	
P1-CTC3	43,01	0,61	*Ni et al. (2013)*	*WXS*	
P1-CTC4	61,44	0,53	*Ni et al. (2013)*	*WXS*	
P1-CTC5	35,46	0,64	*Ni et al. (2013)*	*WXS*	
P1-CTC6	40,83	0,67	*Ni et al. (2013)*	*WXS*	
P1-CTC7	57,23	0,55	*Ni et al. (2013)*	*WXS*	
P1-CTC8	49,03	0,60	*Ni et al. (2013)*	*WXS*	
RC-1	34,63	0,96	*Xu et al. (2012)*	*WXS*	*SRA050201*
RC-2	33,82	0,91	*Xu et al. (2012)*	*WXS*	
RC-3	36,42	0,87	*Xu et al. (2012)*	*WXS*	
RC-4	24,99	0,89	*Xu et al. (2012)*	*WXS*	
RC-5	32,63	0,93	*Xu et al. (2012)*	*WXS*	
RC-6	32,56	0,96	*Xu et al. (2012)*	*WXS*	
RC-7	41,03	0,95	*Xu et al. (2012)*	*WXS*	
RC-8	27,29	0,93	*Xu et al. (2012)*	*WXS*	
RC-9	32,13	0,83	*Xu et al. (2012)*	*WXS*	
RC-10	35,2	0,95	*Xu et al. (2012)*	*WXS*	
RC-11	24,72	0,82	*Xu et al. (2012)*	*WXS*	
RC-12	27,59	0,82	*Xu et al. (2012)*	*WXS*	
RC-13	36,06	0,92	*Xu et al. (2012)*	*WXS*	
RC-14	30,4	0,82	*Xu et al. (2012)*	*WXS*	
RC-15	34,42	0,94	*Xu et al. (2012)*	*WXS*	
RC-16	41,32	0,93	*Xu et al. (2012)*	*WXS*	
RC-17	38,1	0,93	*Xu et al. (2012)*	*WXS*	
RC-18	23,91	0,89	*Xu et al. (2012)*	*WXS*	
RC-19	51,74	0,88	*Xu et al. (2012)*	*WXS*	
RC-20	30,76	0,93	*Xu et al. (2012)*	*WXS*	
RN-1	25,85	0,95	*Xu et al. (2012)*	*WXS*	
RN-2	27,45	0,96	*Xu et al. (2012)*	*WXS*	
RN-3	37,41	0,91	*Xu et al. (2012)*	*WXS*	
RN-4	32,65	0,90	*Xu et al. (2012)*	*WXS*	
RN-5	31,56	0,96	*Xu et al. (2012)*	*WXS*	
BC1	43	0,73	*Wang et al. (2014)*	*WGS*	*SRA053195*
BC2	35	0,78	*Wang et al. (2014)*	*WGS*	
BC3	49	0,89	*Wang et al. (2014)*	*WGS*	
BC4	60	0,82	*Wang et al. (2014)*	*WGS*	
c2	56	0,97	*Wang et al. (2014)*	*WXS*	
c3	59	0,96	*Wang et al. (2014)*	*WXS*	
c4	46	0,96	*Wang et al. (2014)*	*WXS*	
c5	41	0,96	*Wang et al. (2014)*	*WXS*	
c6	39	0,96	*Wang et al. (2014)*	*WXS*	

c7	53	0,97	*Wang et al. (2014)*	*WXS*	
c8	86	0,98	*Wang et al. (2014)*	*WXS*	
c9	63	0,97	*Wang et al. (2014)*	*WXS*	
c10	57	0,97	*Wang et al. (2014)*	*WXS*	
c11	42	0,96	*Wang et al. (2014)*	*WXS*	
c12	110	0,96	*Wang et al. (2014)*	*WXS*	
c13	82	0,95	*Wang et al. (2014)*	*WXS*	
c14	92	0,94	*Wang et al. (2014)*	*WXS*	
c15	162	0,96	*Wang et al. (2014)*	*WXS*	
c16	50	0,92	*Wang et al. (2014)*	*WXS*	
c17	80	0,92	*Wang et al. (2014)*	*WXS*	
c18	31	0,88	*Wang et al. (2014)*	*WXS*	
c19	28	0,88	*Wang et al. (2014)*	*WXS*	
c20	24	0,87	*Wang et al. (2014)*	*WXS*	
c21	25	0,86	*Wang et al. (2014)*	*WXS*	
c22	44	0,95	*Wang et al. (2014)*	*WXS*	
c23	46	0,93	*Wang et al. (2014)*	*WXS*	
c24	53	0,97	*Wang et al. (2014)*	*WXS*	
c25	47	0,93	*Wang et al. (2014)*	*WXS*	
c26	34	0,94	*Wang et al. (2014)*	*WXS*	
c27	35	0,94	*Wang et al. (2014)*	*WXS*	
c28	36	0,94	*Wang et al. (2014)*	*WXS*	
c29	39	0,95	*Wang et al. (2014)*	*WXS*	
c30	38	0,94	*Wang et al. (2014)*	*WXS*	
c31	36	0,94	*Wang et al. (2014)*	*WXS*	
c32	44	0,95	*Wang et al. (2014)*	*WXS*	
c34	38	0,94	*Wang et al. (2014)*	*WXS*	
c35	46	0,94	*Wang et al. (2014)*	*WXS*	
c36	33	0,91	*Wang et al. (2014)*	*WXS*	
c37	39	0,96	*Wang et al. (2014)*	*WXS*	
c38	43	0,96	*Wang et al. (2014)*	*WXS*	
c39	33	0,92	*Wang et al. (2014)*	*WXS*	
c40	40	0,96	*Wang et al. (2014)*	*WXS*	
c41	30	0,94	*Wang et al. (2014)*	*WXS*	
c42	32	0,95	*Wang et al. (2014)*	*WXS*	
c43	32	0,95	*Wang et al. (2014)*	*WXS*	
c44	46	0,94	*Wang et al. (2014)*	*WXS*	
c45	30	0,92	*Wang et al. (2014)*	*WXS*	
c46	35	0,89	*Wang et al. (2014)*	*WXS*	
c47	27	0,90	*Wang et al. (2014)*	*WXS*	
n1	50	0,87	*Wang et al. (2014)*	*WXS*	
n2	46	0,86	*Wang et al. (2014)*	*WXS*	
n3	51	0,89	*Wang et al. (2014)*	*WXS*	
n4	48	0,82	*Wang et al. (2014)*	*WXS*	
n5	55	0,79	*Wang et al. (2014)*	*WXS*	
n7	30	0,89	*Wang et al. (2014)*	*WXS*	
n8	22	0,84	*Wang et al. (2014)*	*WXS*	
n10	34	0,94	*Wang et al. (2014)*	*WXS*	

n11	40	0,93	*Wang et al. (2014)*	*WXS*	
n12	39	0,95	*Wang et al. (2014)*	*WXS*	
LC-1	22,21	0,86	*Hou et al. (2012)*	*WXS*	*SRA050202*
LC-100	40,09	0,93	*Hou et al. (2012)*	*WXS*	
LC-12	25,29	0,79	*Hou et al. (2012)*	*WXS*	
LC-16	37,27	0,88	*Hou et al. (2012)*	*WXS*	
LC-18	27,47	0,80	*Hou et al. (2012)*	*WXS*	
LC-19	26,37	0,72	*Hou et al. (2012)*	*WXS*	
LC-2	31,61	0,84	*Hou et al. (2012)*	*WXS*	
LC-20	31,12	0,78	*Hou et al. (2012)*	*WXS*	
LC-22	34,02	0,90	*Hou et al. (2012)*	*WXS*	
LC-24	25,55	0,82	*Hou et al. (2012)*	*WXS*	
LC-25	27,56	0,86	*Hou et al. (2012)*	*WXS*	
LC-26	35,52	0,77	*Hou et al. (2012)*	*WXS*	
LC-29	27,19	0,84	*Hou et al. (2012)*	*WXS*	
LC-3	33,58	0,83	*Hou et al. (2012)*	*WXS*	
LC-30	24,62	0,82	*Hou et al. (2012)*	*WXS*	
LC-31	30,73	0,75	*Hou et al. (2012)*	*WXS*	
LC-36	32,9	0,72	*Hou et al. (2012)*	*WXS*	
LC-37	31,59	0,78	*Hou et al. (2012)*	*WXS*	
LC-40	26,93	0,83	*Hou et al. (2012)*	*WXS*	
LC-41	9,97	0,76	*Hou et al. (2012)*	*WXS*	
LC-43	32	0,87	*Hou et al. (2012)*	*WXS*	
LC-44	49,37	0,90	*Hou et al. (2012)*	*WXS*	
LC-45	48,26	0,92	*Hou et al. (2012)*	*WXS*	
LC-47	56,24	0,94	*Hou et al. (2012)*	*WXS*	
LC-48	21,22	0,92	*Hou et al. (2012)*	*WXS*	
LC-49	25,86	0,95	*Hou et al. (2012)*	*WXS*	
LC-5	25,53	0,87	*Hou et al. (2012)*	*WXS*	
LC-50	38,2	0,91	*Hou et al. (2012)*	*WXS*	
LC-52	36,39	0,95	*Hou et al. (2012)*	*WXS*	
LC-54	43,65	0,90	*Hou et al. (2012)*	*WXS*	
LC-56	37,3	0,96	*Hou et al. (2012)*	*WXS*	
LC-6	25,58	0,89	*Hou et al. (2012)*	*WXS*	
LC-60	36,72	0,96	*Hou et al. (2012)*	*WXS*	
LC-61	37,81	0,93	*Hou et al. (2012)*	*WXS*	
LC-63	32,96	0,91	*Hou et al. (2012)*	*WXS*	
LC-66	30,79	0,94	*Hou et al. (2012)*	*WXS*	
LC-69	29,06	0,93	*Hou et al. (2012)*	*WXS*	
LC-7	26,59	0,78	*Hou et al. (2012)*	*WXS*	
LC-70	32,38	0,92	*Hou et al. (2012)*	*WXS*	
LC-72	37,46	0,83	*Hou et al. (2012)*	*WXS*	
LC-73	15,46	0,69	*Hou et al. (2012)*	*WXS*	
LC-74	40,39	0,96	*Hou et al. (2012)*	*WXS*	
LC-76	37,59	0,71	*Hou et al. (2012)*	*WXS*	
LC-78	41,25	0,78	*Hou et al. (2012)*	*WXS*	
LC-79	27,74	0,95	*Hou et al. (2012)*	*WXS*	

LC-8	24,64	0,88	*Hou et al. (2012)*	*WXS*	
LC-80	51,5	0,92	*Hou et al. (2012)*	*WXS*	
LC-82	47,35	0,93	*Hou et al. (2012)*	*WXS*	
LC-86	55,62	0,93	*Hou et al. (2012)*	*WXS*	
LC-87	43,93	0,89	*Hou et al. (2012)*	*WXS*	
LC-88	36,68	0,77	*Hou et al. (2012)*	*WXS*	
LC-89	42,65	0,82	*Hou et al. (2012)*	*WXS*	
LC-9	29,81	0,77	*Hou et al. (2012)*	*WXS*	
LC-90	34,64	0,83	*Hou et al. (2012)*	*WXS*	
LC-91	31,66	0,83	*Hou et al. (2012)*	*WXS*	
LC-93	36,82	0,92	*Hou et al. (2012)*	*WXS*	
LC-97	32,45	0,92	*Hou et al. (2012)*	*WXS*	
LN-5	25,37	0,32	*Hou et al. (2012)*	*WXS*	
LN-7	34,54	0,43	*Hou et al. (2012)*	*WXS*	
LN-14	39,44	0,28	*Hou et al. (2012)*	*WXS*	
LN-17	31,16	0,32	*Hou et al. (2012)*	*WXS*	
LN-28	43,77	0,34	*Hou et al. (2012)*	*WXS*	
LN-29	119,4	0,43	*Hou et al. (2012)*	*WXS*	
LN-30	48,9	0,54	*Hou et al. (2012)*	*WXS*	
LN-31	25,74	0,19	*Hou et al. (2012)*	*WXS*	

Table SII. List of previously reported non-synonymous mutations

Chromosome	*Genomic position (Hg19)*	*Gene Name*	*Study*
1	19415329	UBR4	*Ni et al. (2013)*
1	35824705	ZMYM4	*Ni et al. (2013)*
1	199997039	NR5A2	*Ni et al. (2013)*
2	74007164	DUSP11	*Ni et al. (2013)*
2	112933344	FBLN7	*Ni et al. (2013)*
2	125521689	CNTNAP5	*Ni et al. (2013)*
2	179423113	TTN	*Ni et al. (2013)*
2	179596666	TTN	*Ni et al. (2013)*
2	179612324	TTN	*Ni et al. (2013)*
2	206869847	INO80D	*Ni et al. (2013)*
2	209212646	PIKFYVE	*Ni et al. (2013)*
3	27431460	SLC4A7	*Ni et al. (2013)*
3	30691922	TGFBR2	*Ni et al. (2013)*
3	51907931	IQCF5	*Ni et al. (2013)*
3	178936091	PIK3CA	*Ni et al. (2013)*
4	183651411	ODZ3	*Ni et al. (2013)*
5	112179782	APC	*Ni et al. (2013)*
5	127647024	FBN2	*Ni et al. (2013)*
5	141353167	RNF14	*Ni et al. (2013)*
6	31473633	MICB	*Ni et al. (2013)*
6	34835056	UHRF1BP1	*Ni et al. (2013)*
6	35423973	FANCE	*Ni et al. (2013)*
6	52996883	GCM1	*Ni et al. (2013)*
7	110763611	LRRN3	*Ni et al. (2013)*
8	73480124	KCNB2	*Ni et al. (2013)*
9	15203853	TTC39B	*Ni et al. (2013)*
10	94835050	CYP26A1	*Ni et al. (2013)*
10	100174885	PYROXD2	*Ni et al. (2013)*
10	116093049	AFAP1L2	*Ni et al. (2013)*
11	100863155	TMEM133	*Ni et al. (2013)*
11	123484217	GRAMD1B	*Ni et al. (2013)*
11	125888323	CDON	*Ni et al. (2013)*
12	11174534	TAS2R19	*Ni et al. (2013)*
12	57642536	STAC3	*Ni et al. (2013)*
12	81762589	PPFIA2	*Ni et al. (2013)*
12	105600927	APPL2	*Ni et al. (2013)*
13	95095801	DCT	*Ni et al. (2013)*
14	20711536	OR11H4	*Ni et al. (2013)*
14	20760258	TTC5	*Ni et al. (2013)*
14	21875094	CHD8	*Ni et al. (2013)*
15	80445418	FAH	*Ni et al. (2013)*
17	7578466	TP53	*Ni et al. (2013)*
17	33316621	LIG3	*Ni et al. (2013)*
17	42957991	EFTUD2	*Ni et al. (2013)*

17	80202688	CSNK1D	*Ni et al. (2013)*
18	61649004	SERPINB8	*Ni et al. (2013)*
19	49867931	DKKL1	*Ni et al. (2013)*
19	50550285	ZNF473	*Ni et al. (2013)*
20	62195987	PRIC285	*Ni et al. (2013)*
X	55479352	MAGEH1	*Ni et al. (2013)*
X	102931628	MORF4L2	*Ni et al. (2013)*
X	123519644	ODZ1	*Ni et al. (2013)*
X	132887970	GPC3	*Ni et al. (2013)*
X	148797710	MAGEA11	*Ni et al. (2013)*
1	161681812	FCRLA	*Xu et al. (2012)*
1	67206387	SGIP1	*Xu et al. (2012)*
1	9097665	SLC2A5	*Xu et al. (2012)*
1	210010179	DIEXF	*Xu et al. (2012)*
1	192545961	RGS1	*Xu et al. (2012)*
1	1455652	ATAD3A	*Xu et al. (2012)*
1	17272075	CROCC	*Xu et al. (2012)*
1	34038255	CSMD2	*Xu et al. (2012)*
1	89448812	RBMXL1	*Xu et al. (2012)*
2	45789811	SRBD1	*Xu et al. (2012)*
2	130952038	TUBA3E	*Xu et al. (2012)*
2	234638394	UGT1A3	*Xu et al. (2012)*
2	228221804	MFF	*Xu et al. (2012)*
2	234638445	UGT1A3	*Xu et al. (2012)*
3	9066948	SRGAP3	*Xu et al. (2012)*
3	108474687	RETNLB	*Xu et al. (2012)*
4	151520231	LRBA	*Xu et al. (2012)*
4	138450875	PCDH18	*Xu et al. (2012)*
4	41984794	DCAF4L1	*Xu et al. (2012)*
5	36961650	NIPBL	*Xu et al. (2012)*
5	68498375	CENPH	*Xu et al. (2012)*
5	70927955	MCCC2	*Xu et al. (2012)*
5	42801426	SEPP1	*Xu et al. (2012)*
5	75914104	F2RL2	*Xu et al. (2012)*
5	150704907	SLC36A2	*Xu et al. (2012)*
6	10586542	GCNT2	*Xu et al. (2012)*
6	39325092	KIF6	*Xu et al. (2012)*
6	31322911	HLA-B	*Xu et al. (2012)*
6	31322910	HLA-B	*Xu et al. (2012)*
6	33037639	HLA-DPA1	*Xu et al. (2012)*
6	30692160	TUBB	*Xu et al. (2012)*
6	167791549	TCP10	*Xu et al. (2012)*
6	161054929	LPA	*Xu et al. (2012)*
7	16900147	AGR3	*Xu et al. (2012)*
7	144061222	ARHGEF5	*Xu et al. (2012)*
7	5330383	SLC29A4	*Xu et al. (2012)*
7	33015987	FKBP9	*Xu et al. (2012)*

7	149191506	ZNF746	*Xu et al. (2012)*
7	44046934	SPDYE1	*Xu et al. (2012)*
8	68942885	PREX2	*Xu et al. (2012)*
8	54141861	OPRK1	*Xu et al. (2012)*
8	135614406	ZFAT	*Xu et al. (2012)*
8	146016704	RPL8	*Xu et al. (2012)*
8	87060914	PSKH2	*Xu et al. (2012)*
8	67789630	C8orf45	*Xu et al. (2012)*
8	101727750	PABPC1	*Xu et al. (2012)*
9	109688470	ZNF462	*Xu et al. (2012)*
9	3262985	RFX3	*Xu et al. (2012)*
10	25887635	GPR158	*Xu et al. (2012)*
10	22880652	PIP4K2A	*Xu et al. (2012)*
10	124594540	CUZD1	*Xu et al. (2012)*
10	111642207	XPNPEP1	*Xu et al. (2012)*
11	62285556	AHNAK	*Xu et al. (2012)*
11	6292615	CCKBR	*Xu et al. (2012)*
11	118255625	UBE4A	*Xu et al. (2012)*
12	51388333	SLC11A2	*Xu et al. (2012)*
12	133503641	ZNF605	*Xu et al. (2012)*
12	117696917	NOS1	*Xu et al. (2012)*
12	100731284	SCYL2	*Xu et al. (2012)*
12	40699689	LRRK2	*Xu et al. (2012)*
12	11506837	PRB1	*Xu et al. (2012)*
14	94756741	SERPINA10	*Xu et al. (2012)*
14	20711215	OR11H4	*Xu et al. (2012)*
15	45007836	B2M	*Xu et al. (2012)*
15	74363764	GOLGA6A	*Xu et al. (2012)*
15	21066520	POTEB	*Xu et al. (2012)*
16	66420923	CDH5	*Xu et al. (2012)*
16	70729468	VAC14	*Xu et al. (2012)*
16	74444923	CLEC18B	*Xu et al. (2012)*
16	28617413	SULT1A1	*Xu et al. (2012)*
17	60060330	MED13	*Xu et al. (2012)*
17	78321468	RNF213	*Xu et al. (2012)*
17	34581473	TBC1D3F	*Xu et al. (2012)*
17	19476142	SLC47A1	*Xu et al. (2012)*
17	62892031	LRRC37A3	*Xu et al. (2012)*
17	45219615	CDC27	*Xu et al. (2012)*
17	263367	C17orf97	*Xu et al. (2012)*
17	33520392	AMAC1	*Xu et al. (2012)*
17	5036224	USP6	*Xu et al. (2012)*
17	74288567	QRICH2	*Xu et al. (2012)*
19	10116301	COL5A3	*Xu et al. (2012)*
19	54705126	RPS9	*Xu et al. (2012)*
19	42315271	CEACAM3	*Xu et al. (2012)*
19	4363791	SH3GL1	*Xu et al. (2012)*
19	54725930	LILRB3	*Xu et al. (2012)*

19	58265519	ZNF776	*Xu et al. (2012)*
19	8668659	ADAMTS10	*Xu et al. (2012)*
19	15918177	OR10H1	*Xu et al. (2012)*
19	41354171	CYP2A6	*Xu et al. (2012)*
20	41408867	PTPRT	*Xu et al. (2012)*
21	15537643	LIPI	*Xu et al. (2012)*
21	22656592	NCAM2	*Xu et al. (2012)*
22	25023893	GGT1	*Xu et al. (2012)*
22	29454821	C22orf31	*Xu et al. (2012)*
22	25011062	GGT1	*Xu et al. (2012)*
22	39496328	APOBEC3H	*Xu et al. (2012)*
1	172011274	DNM3	*Wang et al. (2014)*
1	248039252	TRIM58	*Wang et al. (2014)*
3	112190167	BTLA	*Wang et al. (2014)*
3	125690977	ROPN1B	*Wang et al. (2014)*
3	178952085	PIK3CA	*Wang et al. (2014)*
3	194392797	LSG1	*Wang et al. (2014)*
4	185553463	CASP3	*Wang et al. (2014)*
5	127680113	FBN2	*Wang et al. (2014)*
10	3214939	PITRM1	*Wang et al. (2014)*
11	72700016	FCHSD2	*Wang et al. (2014)*
14	63888761	PPP2R5E	*Wang et al. (2014)*
X	27998907	DCAF8L1	*Wang et al. (2014)*
1	117142700	IGSF3	*Hou et al. (2012)*
1	117146592	IGSF3	*Hou et al. (2012)*
1	144955288	PDE4DIP	*Hou et al. (2012)*
1	148579636	NBPF15	*Hou et al. (2012)*
1	156843542	NTRK1	*Hou et al. (2012)*
1	28598287	SESN2	*Hou et al. (2012)*
1	36552858	TEKT2	*Hou et al. (2012)*
2	112614429	ANAPC1	*Hou et al. (2012)*
2	131103405	IMP4	*Hou et al. (2012)*
2	233704620	GIGYF2	*Hou et al. (2012)*
2	240982257	PRR21	*Hou et al. (2012)*
2	71649966	ZNF638	*Hou et al. (2012)*
2	85570849	RETSAT	*Hou et al. (2012)*
2	88083761	RGPD1	*Hou et al. (2012)*
3	124390722	KALRN	*Hou et al. (2012)*
3	38149209	DLEC1	*Hou et al. (2012)*
3	47451778	PTPN23	*Hou et al. (2012)*
4	190881979	FRG1	*Hou et al. (2012)*
4	57340538	SRP72	*Hou et al. (2012)*
5	118506685	DMXL1	*Hou et al. (2012)*
5	150028952	SYNPO	*Hou et al. (2012)*
5	150632832	GM2A	*Hou et al. (2012)*
5	178634683	ADAMTS2	*Hou et al. (2012)*

6	30610758	ATAT1	*Hou et al. (2012)*
6	30862440	DDR1	*Hou et al. (2012)*
6	31239108	HLA-C	*Hou et al. (2012)*
6	31322910	HLA-B	*Hou et al. (2012)*
6	32610009	HLA-DQA1	*Hou et al. (2012)*
6	32629935	HLA-DQB1	*Hou et al. (2012)*
6	34574487	C6orf106	*Hou et al. (2012)*
7	143417083	FAM115C	*Hou et al. (2012)*
7	144059791	ARHGEF5	*Hou et al. (2012)*
7	151932945	MLL3	*Hou et al. (2012)*
7	151932997	MLL3	*Hou et al. (2012)*
7	20762646	ABCB5	*Hou et al. (2012)*
7	97493705	ASNS	*Hou et al. (2012)*
8	101727792	PABPC1	*Hou et al. (2012)*
8	101727801	PABPC1	*Hou et al. (2012)*
8	10583909	SOX7	*Hou et al. (2012)*
8	11189616	AMAC1L2	*Hou et al. (2012)*
8	121061879	DEPTOR	*Hou et al. (2012)*
8	144398191	TOP1MT	*Hou et al. (2012)*
8	144620342	ZC3H3	*Hou et al. (2012)*
8	144621084	ZC3H3	*Hou et al. (2012)*
8	144623574	ZC3H3	*Hou et al. (2012)*
8	145006674	PLEC	*Hou et al. (2012)*
9	35811508	SPAG8	*Hou et al. (2012)*
9	43129545	ANKRD20A2	*Hou et al. (2012)*
9	69423637	ANKRD20A4	*Hou et al. (2012)*
10	74896664	ECD	*Hou et al. (2012)*
12	11546380	PRB2	*Hou et al. (2012)*
12	48359984	TMEM106C	*Hou et al. (2012)*
12	52708420	KRT83	*Hou et al. (2012)*
12	52865925	KRT6C	*Hou et al. (2012)*
12	53671005	ESPL1	*Hou et al. (2012)*
14	32561340	ARHGAP5	*Hou et al. (2012)*
14	39819314	CTAGE5	*Hou et al. (2012)*
14	95916404	C14orf49	*Hou et al. (2012)*
15	41060179	DNAJC17	*Hou et al. (2012)*
16	3119304	IL32	*Hou et al. (2012)*
16	68265239	ESRP2	*Hou et al. (2012)*
17	20363677	LGALS9B	*Hou et al. (2012)*
17	33520392	AMAC1	*Hou et al. (2012)*
17	39197477	KRTAP1-1	*Hou et al. (2012)*
17	58288421	USP32	*Hou et al. (2012)*
17	62893332	LRRC37A3	*Hou et al. (2012)*
18	43833701	C18orf25	*Hou et al. (2012)*
19	41622107	CYP2F1	*Hou et al. (2012)*
19	54726833	LILRB3	*Hou et al. (2012)*
19	58991900	ZNF446	*Hou et al. (2012)*
19	1004740	GRIN3B	*Hou et al. (2012)*

20	44515559	C20orf165	*Hou et al. (2012)*
20	62197300	PRIC285	*Hou et al. (2012)*
21	47614469	LSS	*Hou et al. (2012)*
22	21355576	THAP7	*Hou et al. (2012)*
22	29704343	GAS2L1	*Hou et al. (2012)*
22	41222607	ST13	*Hou et al. (2012)*
22	50689427	HDAC10	*Hou et al. (2012)*

Supplementary Figures

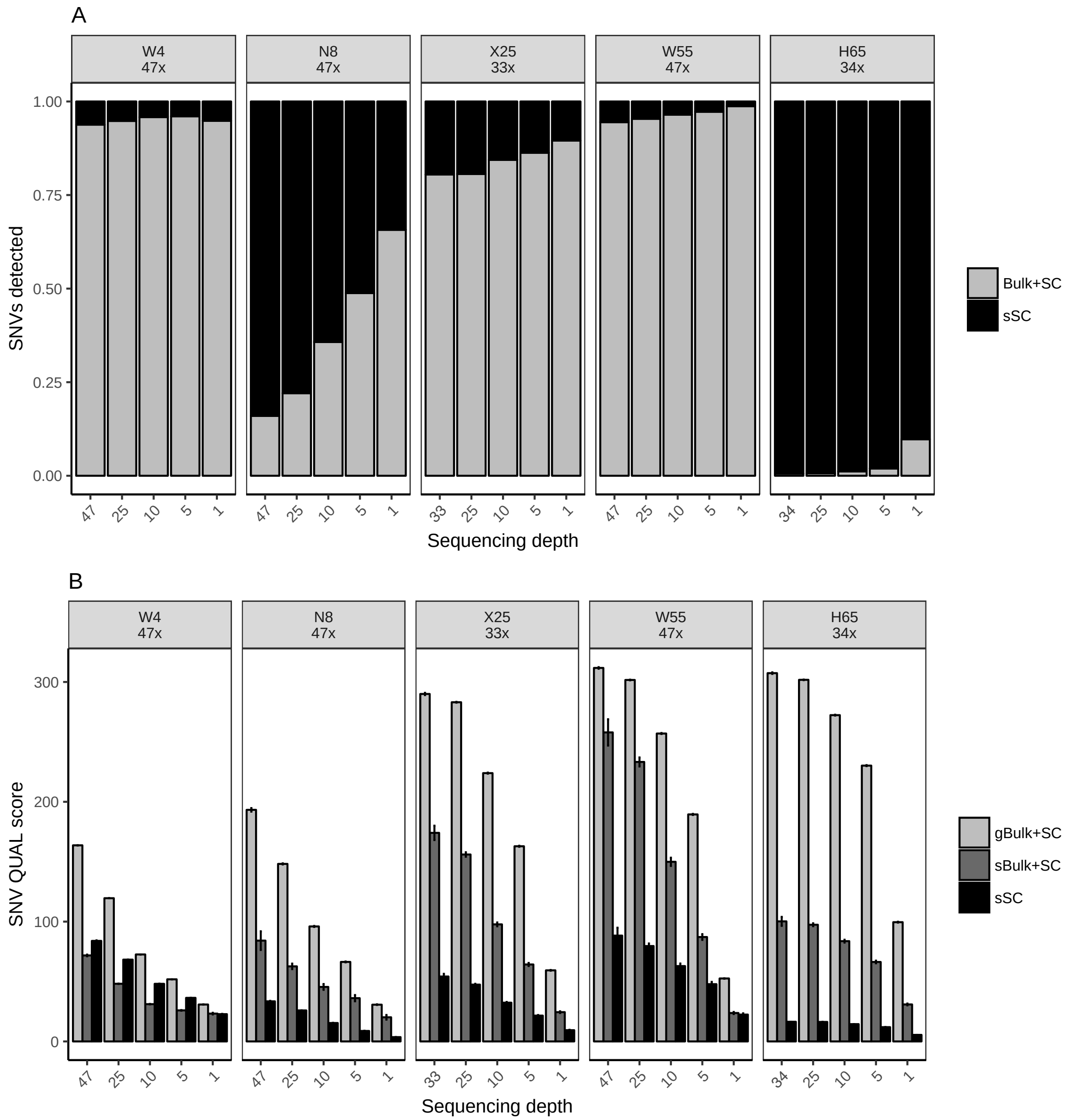

Figure S1. Single-cell specific SNVs.

(A) Barplots illustrating the proportion of germline and somatic SNVs detected in bulk and SC-Seq datasets (Bulk +SC) versus variants exclusively found in single-cell datasets (sSC). (B) Barplots displaying the variant quality scores for different variant "types": gBulk+SC refers to germline variants observed in both bulk and single-cell datasets; sBulk+SC refers to somatic variants observed in both bulk and single-cell datasets; sSC corresponds to variants called solely in single-cell datasets.

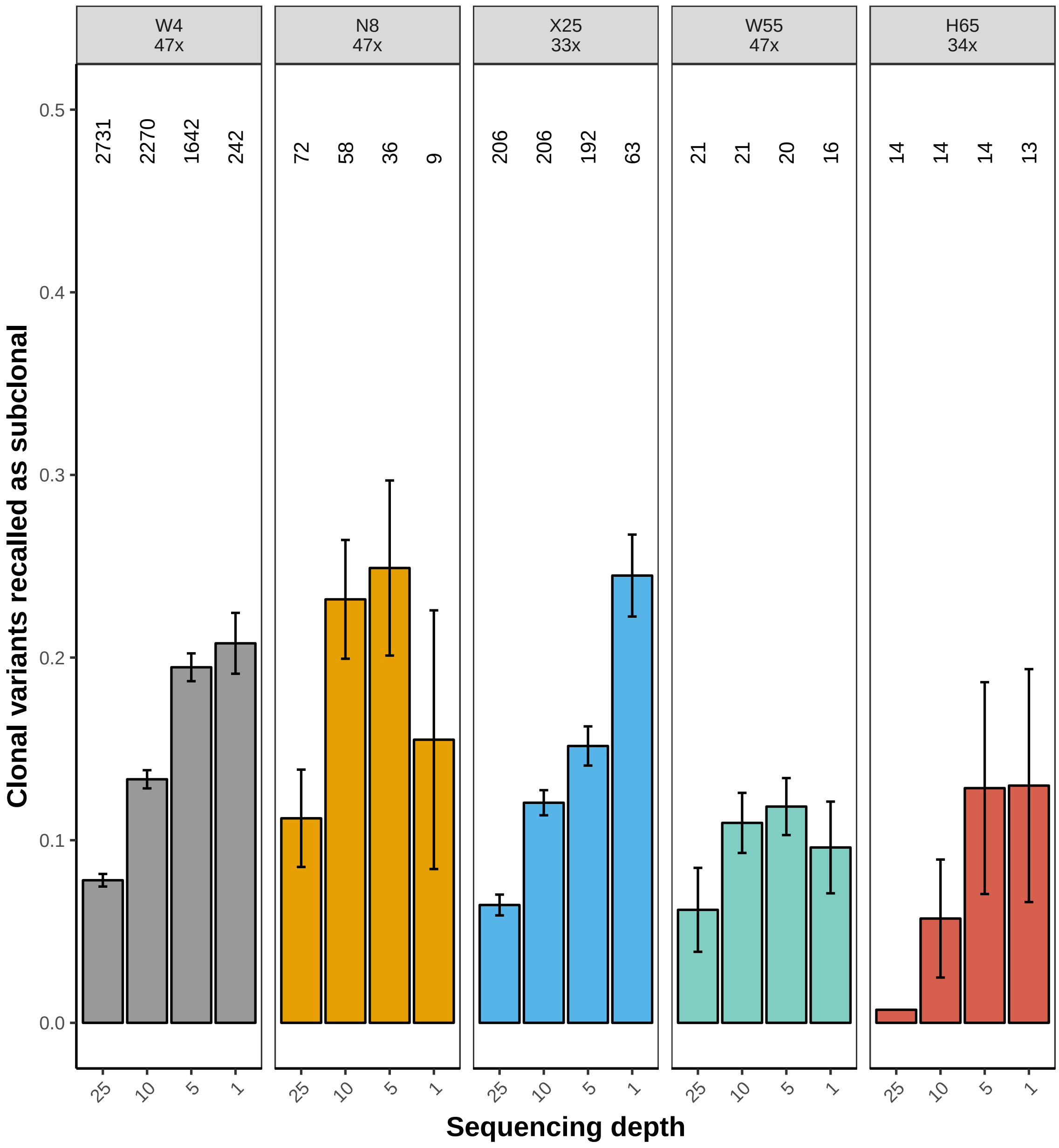

Figure S2. Clonal and subclonal SNV recall.

Barplots showing the proportion of clonal variants identified in the original datasets that were recalled as subclonal in the down-sampled datasets. Numbers above bars indicate the absolute number of clonal variants ascertained in original dataset that were also present in the down-sampled datasets. Error bars indicate 95% confidence intervals.

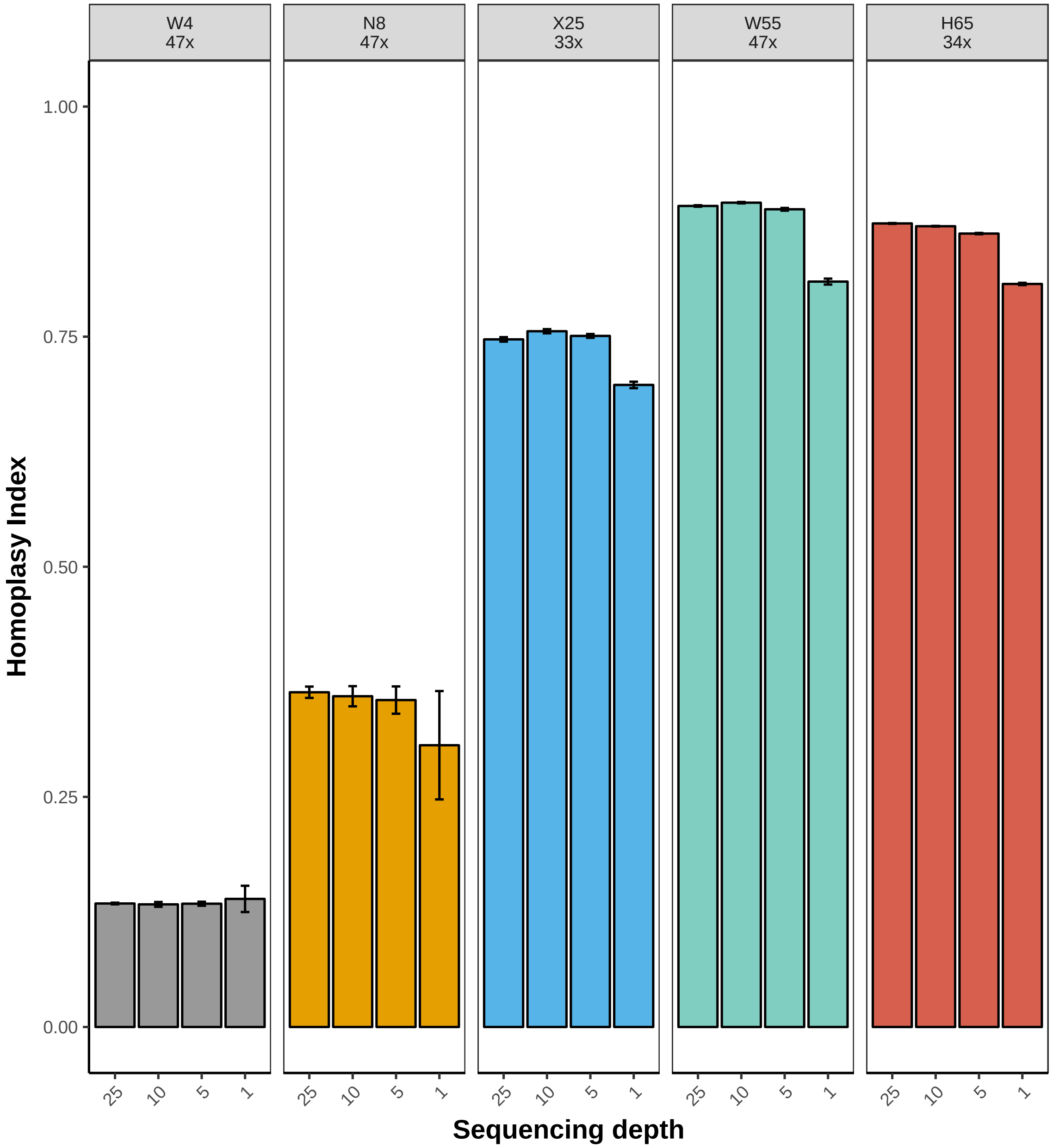

Figure S3. Phylogenetic Tree consistency.

Barplots displaying the Homoplasy Index (HI) across the different sequencing depths. Error bars indicate 95% confidence intervals.
